# The National Burden of Colorectal Cancer in the United States from 1990 to 2019

**DOI:** 10.3390/cancers16010205

**Published:** 2024-01-01

**Authors:** Saqr Alsakarneh, Fouad Jaber, Azizullah Beran, Mohammad Aldiabat, Yazan Abboud, Noor Hassan, Mohamed Abdallah, Thaer Abdelfattah, Laith Numan, Wendell Clarkston, Mohammad Bilal, Aasma Shaukat

**Affiliations:** 1 Department of Internal Medicine, University of Missouri, Kansas City, MO 64110, USA; fouad.jaber@umkc.edu (F.J.); noorhassan@umkc.edu (N.H.); 2 Division of Gastroenterology and Hepatology, Indiana University School of Medicine, Indianapolis, IN 46202, USA; aziz@iu.edu; 3 Harvard T.H. Chan School of Public Health, Harvard University, Boston, MA 02115, USA; maldiabat@hsph.harvard.edu; 4 Department of Internal Medicine, Rutgers New Jersey Medical School, Newark, NJ 07013, USA; ya296@njms.rutgers.edu; 5 Division of Gastroenterology and Hepatology, Department of Medicine, University of Minnesota, Minneapolis, MN 55455, USA; mohamedabdallah112@gmail.com; 6 Division of Gastroenterology and Hepatology, Allegheny Health Network, Pittsburgh, PA 15212, USA; thaer.abdelfattah@gmail.com; 7 Division of Gastroenterology and Hepatology, Saint Louis University, St. Louis, MO 63103, USA; laith.numan90@gmail.com; 8 Division of Gastroenterology and Hepatology, University of Missouri, Kansas City, MO 64110, USA; clarkstonw@umkc.edu; 9 Division of Gastroenterology and Hepatology, Minneapolis Veterans Affairs Health Care System, Minneapolis, MN 55417, USA; billa17@hotmail.com; 10 Division of Gastroenterology, Department of Medicine and Population Health, Grossman School of Medicine, New York University, New York, NY 10003, USA; aasma.shaukat@nyulangone.org

**Keywords:** colorectal cancer, epidemiology, trends, health disparity, colon cancer

## Abstract

**Simple Summary:**

Colorectal cancer (CRC) is a significant global health concern, ranking as the second leading cause of cancer-related deaths. Despite an overall decline in CRC rates in the United States, there is a concerning rise in early-onset cases (diagnosed before age 50). This study, utilizing comprehensive data from the Global Burden of Disease 2019, delves into the state-specific trends in CRC incidence, prevalence, and mortality over three decades (1990–2019). Notably, the research reveals varying patterns across geographic regions, gender, and age groups. Understanding these nuanced trends is crucial for targeted resource allocation and policy decisions to effectively combat CRC, a disease with an increasing impact on younger adults in the United States.

**Abstract:**

CRC accounts for approximately a tenth of all cancer cases and deaths in the US. Due to large differences in demographics among the different states, we aim to determine trends in the CRC epidemiology and across different states, age groups, and genders. CRC rates, age-adjusted to the standard US population, were obtained from the GBD 2019 database. Time trends were estimated as annual percentage change (APC). A pairwise comparison was conducted between age- and gender-specific trends using the tests of parallelism and coincidence. Age-specific trends were also assessed in two age subgroups: younger adults aged 15–49 years and older adults aged 50–74 years. We also analyzed the prevalence, incidence, mortality, and DALYs in the US between 1990 and 2019. A total of 5.53 million patients were diagnosed with CRC in the US between 1990 and 2019. Overall, CRC incidence rates have significantly increased in younger adults (11.1 per 100,000 persons) and decreased in older adults (136.8 per 100,000 persons) (AAPC = 1.2 vs. −0.6; AAPC difference = 1.8, *p* < 0.001). Age-specific trends were neither identical (*p* < 0.001) nor parallel (*p* < 0.001), suggesting that CRC incidence rates are different and increasing at a greater rate in younger adults compared to older adults. However, for both men and women (49.4 and 35.2 per 100,000 persons), incidence rates have decreased over the past three decades at the same rate (AAPC = −0.5 vs. −0.5; AAPC difference = 0, *p* = 0.1). Geographically, the southern states had the highest mortality rates with Mississippi having the highest rate of 20.1 cases per 100,000 population in 2019. Massachusetts, New York, and the District of Colombia had the greatest decreases in mortality over the study period (−42.1%, −41.4%, and −40.9%). Decreased mortality was found in all states except Mississippi, where the mortality of CRC increased over the study period (+1.5%). This research provides crucial insights for policymakers to tailor resource allocation, emphasizing the dynamic nature of CRC burden across states and age groups, ultimately informing targeted strategies for prevention and intervention.

## 1. Introduction

Colorectal cancer (CRC) accounts for approximately a tenth of all cancer cases and deaths worldwide [1]. The incidence of CRC ranks third among all cancers, whereas CRC is the second most common cause of cancer-related mortality [2]. The incidence of and mortality from CRC are increasing worldwide, with estimates to exceed 2.2 million new cases and 1.1 million deaths by 2030 [3,4]. While the overall incidence of CRC has declined in the United States (US), there has been a steady increase in early-onset CRC diagnosed before the age of 50 since the 1980s [5]. This has resulted in a substantial cancer burden in younger adults [6].

CRC burden can vary considerably by geographic location, gender, and age group [6]. Accurately assessing the incidence, prevalence, and mortality rates posed by CRC in these demographics remains an ongoing challenge. Despite various studies offering valuable insights and knowledge into CRC by studying data at a regional, national, and multi-country level, there remains a paucity of comprehensive information regarding the disease burden specific to geographic location, gender, and age at a more granular state level within the US [6,7]. This information is valuable in order to appropriately and effectively allocate both medical and financial resources throughout the country.

The Global Burden of Disease (GBD) and Injuries and Risk Factors Study 2019 (GBD 2019) framework provides data on incident cases, deaths, and DALYs of 369 diseases and injuries in 204 countries. While the CRC burden is well elucidated based on GBD 2019 [6,8,9,10,11], the available information on its effect on individual states and the recent disease burden trends are notably deficient. Therefore, we aimed to provide a systematic report of the CRC burden, including incidence, prevalence, mortality, and DALYs at a state level in the US. Additionally, we aimed to study age- and gender-specific incidence, prevalence, and mortality rate trends in CRC in the US for the past three decades, from 1990 to 2019, using the GBD 2019 database.

## 2. Materials and Methods

### 2.1. Background

A population-based time-trend analysis of CRC incidence, prevalence, and mortality rates in the US from 1990 to 2019 was conducted using the GBD 2019 study database. This is a publicly accessible database containing anonymized de-identifiable data. Therefore, this study was exempted from review by the institutional review board based on the recommendations of the National Human Research Protections Advisory Committee policy.

### 2.2. Data Source

CRC incidence, prevalence, and death rates in the US between 1990 and 2019 were gathered from the GBD 2019 study database. The GBD 2019 methodically and comprehensively evaluated 286 causes of death, 369 illnesses and injuries, and 87 risk factors from various relevant data sources, including household surveys, censuses, vital statistics, and civil registrations, for 204 nations and territories. The GBD 2019 study was conducted by the Institute for Health Metrics and Evaluation (IHME) at the University of Washington. The GBD study provides a comprehensive and comparable analysis of various health metrics, such as incidence, prevalence, death, years of life lost (YLLs), years lived with disability (YLDs), and DALYs, for various diseases and injuries, as well as demographic and geographical variations.

Data sources and methods for the GBD 2019 study estimates have previously been outlined in detail [12,13,14]. Briefly, structured literature reviews were conducted to find published and unpublished incidence, prevalence, case fatality, and mortality data associated with CRC. The International Classification of Diseases (ICD)-9 and ICD-10 codes for each cause of CRC were obtained. ICD-9 and 10 codes (ICD-10: C18–C21.9, D01.0–D01.3, D12–D12.9, D37.3–D37.5) (ICD-9: 153–154.9, 209.1, 209.5, 211.3–211.4, 230.3–230.6, 569.0) were obtained for each CRC case definition and used to determine the yearly incidence, prevalence, and mortality rate for chosen health conditions, stratified by age, gender, year, and state. The Cause of Death Ensemble model (CODEm), spatiotemporal Gaussian process regression (ST-GPR), and the Bayesian meta-regression tool DisMod-MR were the main methods used to estimate the prevalence, incidence, deaths, YLLs, YLDs, and DALYs by cause, age, sex, year, and location for the GBD 2019 study [13]. 

Meticulous checks and verification ensured the data quality at many department levels, producing highly reliable data. The GBD 2019 Data Input Sources Tool webpage contains detailed information on the original data sources utilized in the present study (http://ghdx.healthdata.org/gbd-2019/data-input-sources (accessed on 25 March 2023)).

### 2.3. Definitions

The number of patients diagnosed with CRC per 100,000 in a particular calendar year was described as the incidence rate. The mortality rate was defined as the number of deaths per 100,000 population attributable to CRC in a particular calendar year. The annual percentage change (APC) was defined as the percentage change in CRC incidence, prevalence, or mortality rates between consecutive years, whereas the average annual percentage change (AAPC) was defined as the mean percentage change per year for the whole study period. Increasing and decreasing trends were defined as statistically significant positive and negative values of APC or AAPC, respectively, while stable trends were regarded as not statistically significant values. The population was divided into two age categories based on a 50-year cutoff: older adults (50–79 years) and younger adults (15–49 years).

### 2.4. Analysis

The Joinpoint Regression Program, v5.0.2 (National Cancer Institute “NCI”), which builds best-fit models for a sequence of logarithmic data, was used to quantify temporal trends [15]. The joinpoint regression model, a collection of linear statistical models, was employed to assess the temporal trends in disease burdens associated with CRC. This model utilizes the least squares method to estimate the changing patterns of illness rates, thus mitigating the subjectivity inherent in conventional trend analyses based on linear trends. By calculating the sum of squared residuals between the estimated and actual values, the joinpoint regression model identifies the turning point where the trend shifts [16].

The analysis was performed with a minimal number of joinpoints (zero, indicating a straight line), followed by model-fitting tests with a maximum of five joinpoints. The program employs Monte Carlo permutation analysis to determine the minimum number of joinpoints required to construct a segmented line that depicts time-dependent change [16]. The APC and AAPC were computed using parametric estimates and a two-sided t-test to determine statistical significance [17]. A pairwise comparison was performed to determine parallelism and homogeneity. The parallelism test determines if the two segmented linear regression mean functions are parallel. This test is conducted on the log-transformed scale of the APCs, and the results are back-transformed to the original scale in the Joinpoint Regression Program’s final output. The statistical significance of the absolute difference between the AAPCs was estimated using a Taylor series expansion. A *p*-value less than 0.05 was considered statistically significant in all analyses.

## 3. Results

### 3.1. Colorectal Cancer Burden in the United States

In the US, the absolute number of all-age deaths attributed to colorectal cancer (CRC) increased from 65,582 (95% CI: 61,888–67,693) in 1990 to 84,026 (95% CI: 77,987–87,516) in 2019, with a relative growth of 28.1%. However, the age-standardized mortality rate (ASMRs) fell by 26.3% for both genders. Similarly, age-standardized DALYs decreased for both genders, with a larger decrease for females (−24.7% vs. −23.8%). Conversely, the age-standardized incidence rate (ASIRs) for CRC decreased more in males than females between 1990 and 2019, with percentage changes of −14.1% and −12.6%, respectively. Age-standardized prevalence rate (ASPRs) decreased from 273.3 (95% Cl: 264.1–281.9) in 1990 to 259.2 (95% Cl: 225.2–297.5) in 2019. Although all absolute numbers increased over the study period, age-standardized incidence, prevalence, and mortality rates decreased between 1990 and 2019 (Supplementary Table S1).

### 3.2. Colorectal Cancer Age-Standardized Incidence Rate with Geographic, Gender, and Age-Specific Variations

#### 3.2.1. Colorectal Cancer Age-Standardized Incidence Rate with Geographic Variation

**Figure 1:**
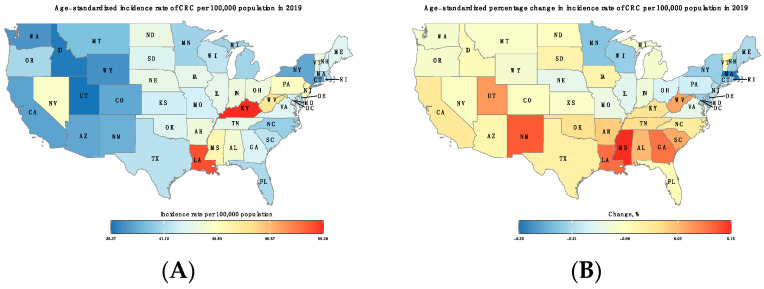
(**A**) Age-standardized incidence rate of CRC per 100,000 population in 2019. (**B**) Percentage change (%) in age-standardized incidence rates of CRC between 1990 and 2019.

Overall, the ASIRs declined in most states from 1990 to 2019. ASIRs dropped by 12.1% (95% CI: 11–13%) in the US with the most significant drop achieved in Massachusetts (−32.9%; 95% CI: 31–35%). Only nine states recorded an increase in ASIRs with the highest increase observed in Mississippi, 14.8% (95% CI: 13–17%). In both 1990 and 2019, several western states were observed to have the lowest ASIRs, with Utah having the lowest ASIRs in both years, 34.4 (95% Cl: 32.1–36.5) and 36.4 (95% Cl: 28.9–45.2), respectively. On the other hand, the highest ASIR in 2019 was observed in Kentucky (55.3; 95% Cl: 44.4–67.3) (Figure 1 and Supplementary Table S2).

#### 3.2.2. Colorectal Cancer Age-Standardized Incidence Rate with Gender Variation

**Figure 2:**
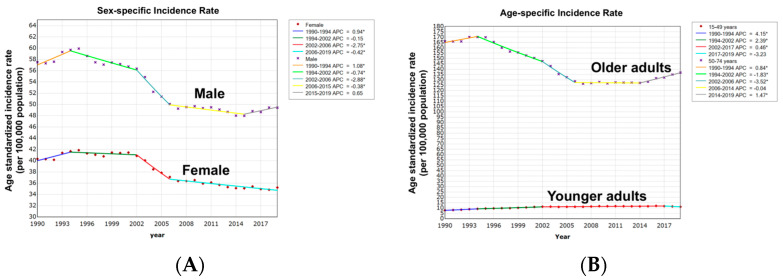
(**A**) Sex-specific trends in CRC age-standardized incidence rates. (**B**) Age-specific trends in CRC age-standardized incidence rates. * = Statistically significant trend (*p* < 0.05).

Over 30 years (1990–2019), there was an overall decrease in ASIRs among men and women at the same rate (AAPC, −0.5%; 95% CI: −0.6% to −0.4%; *p* < 0.001) without significant difference (*p* = 0.46). The most significant drop in ASIRs in both males and females was observed in the 2002–2006 period with an APCs of (−2.88, *p* < 0.001) and (−2.75, *p* < 0.001), respectively (Figure 2 and Supplementary Table S5).

#### 3.2.3. Colorectal Cancer Age-Standardized Incidence Rate with Age Variation

Overall, age-specific ASIRs decreased in older adults (AAPC, −0.6%; 95% CI: −0.7% to −0.5%; *p* < 0.001) and increased in younger adults (AAPC, 1.2%; 95% CI: 1.1% to 1.3%; *p* < 0.001) with an absolute AAPC difference of 1.8% (95% CI: 1.6–2.0%; *p* < 0.001). Age-specific trends were non-identical (*p* < 0.001) and not parallel (*p* < 0.001), suggesting that ASIRs are different and decreasing at a greater rate in younger adults compared to older adults (Figure 2 and Supplementary Table S5).

### 3.3. Colorectal Cancer Age-Standardized Prevalence Rate with Geographic, Gender, and Age-Specific Variations

#### 3.3.1. Colorectal Cancer Age-Standardized Prevalence Rate with Geographic Variation

**Figure 3:**
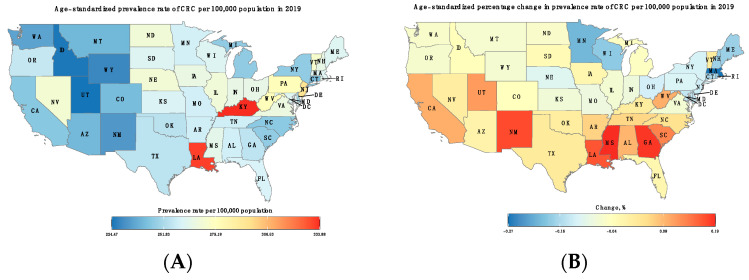
(**A**) Age-standardized prevalence rate of CRC per 100,000 population in 2019. (**B**) Percentage change (%) in age-standardized prevalence rates of CRC between 1990 and 2019.

In 2019, there was a total of 1,376,788 (95% CI: 1,200,009, 1,577,837) patients (45.5% females) diagnosed with CRC in the US. Similar to ASIRs, ASPRs showed wide geographical variations in 2019, with the highest ASPRs also observed in Kentucky (333.9; 95% CI: 269.6–405.6) and the lowest rates observed in many western states, including Utah, Idaho, and Wyoming. Similar to incidence, the largest relative percentage decrease in ASPRs was achieved in Massachusetts (−27.3%). Several states saw an increase in their ASPRs, with the highest relative growth change in Mississippi (19.5%) and Georgia (18.9%) (Figure 3 and Supplementary Table S3).

#### 3.3.2. Colorectal Cancer Age-Standardized Prevalence Rate with Gender Variation

**Figure 4:**
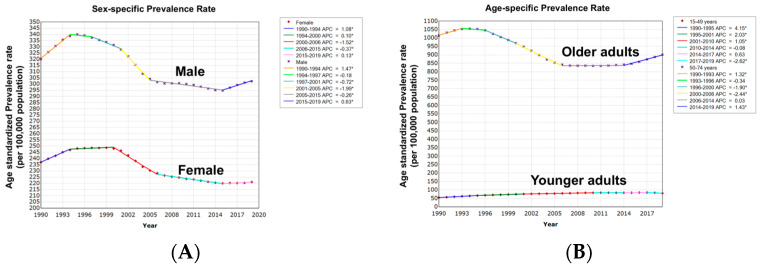
(**A**) Sex-specific trends in CRC age-standardized prevalence rates. (**B**) Age-specific trends in CRC age-standardized prevalence rates. * = Statistically significant trend (*p* < 0.05).

From 1990 to 2019, there was an overall decrease in ASPRs among men and women at the same rate (AAPC, −0.2%; 95% CI: −0.3% to −0.1%; *p* < 0.001) without significant difference (*p* = 0.52). Interestingly, from 2015, there was an overall increase in ASPRs among men (APC = 0.6, *p* < 0.001) (Figure 4 and Supplementary Table S6).

#### 3.3.3. Colorectal Cancer Age-Standardized Prevalence Rate with Age Variation

While older adults had a decrease in ASPRs (AAPC = −0.4%; 95% CI: −0.5% to −0.3%; *p* < 0.001), younger adults had an overall increase in ASPRs (AAPC = 1.3%; 95% CI: 1.1% to 1.5%; *p* < 0.001) with an absolute AAPC difference of 1.7% (95% CI: 1.5–1.9%; *p* < 0.001). Age-specific trends were non-identical (*p* < 0.001) and not parallel (*p* < 0.001), suggesting that ASPRs among younger adults are different and relatively increasing at a greater rate than older adults (Figure 4 and Supplementary Table S6).

### 3.4. Colorectal Cancer Age-Standardized Mortality Rate with Geographic, Gender, and Age-Specific Variations

#### 3.4.1. Colorectal Cancer Age-Standardized Mortality Rate with Geographic Variation

**Figure 5:**
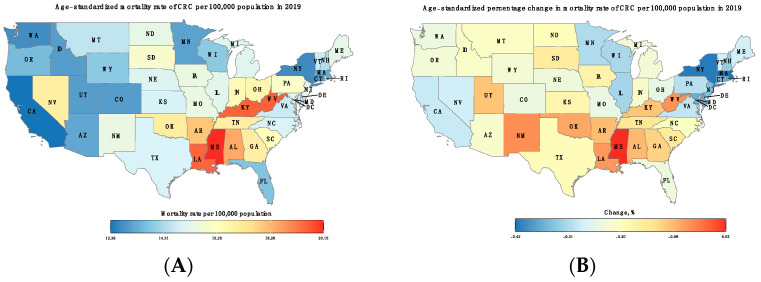
(**A**) Age-standardized mortality rate of CRC per 100,000 population in 2019. (**B**) Percentage change (%) in age-standardized mortality rates of CRC between 1990 and 2019.

The absolute number of deaths attributable to CRC in the U.S. increased from 65,582 (95% CI: 61,888–67,693) in 1990 to 84,026 (95% CI: 77,987–87,516) in 2019. There was a wide geographical variation in ASMRs, with the highest rate recorded in the southern states, including Mississippi, 20.1 per 100,000, and West Virginia, 19.6 per 100,000, followed by Kentucky and Louisiana, 19.5 per 100,000. On the other hand, the lowest ASMRs were observed in California and New York. Interestingly, all states observed a decrease in ASMRs between 1990 and 2019, except for Mississippi, which showed a relative growth of 1.8%. The greatest relative reductions in ASMRs were achieved by Massachusetts, −42.1%, New York, −41.4%, and the District of Columbia, −40.9% (Figure 5 and Supplementary Table S4). 

#### 3.4.2. Colorectal Cancer Age-Standardized Mortality Rate with Gender Variation

**Figure 6:**
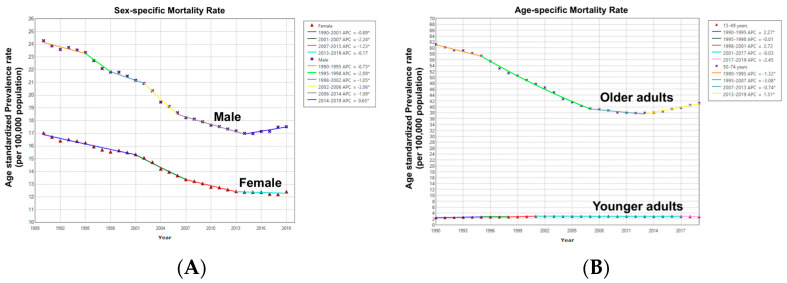
(**A**) Sex-specific trends in CRC age-standardized mortality rates. (**B**) Age-specific trends in CRC age-standardized mortality rates. * = Statistically significant trend (*p* < 0.05).

Overall, ASMRs were similar for men and women (AAPC = −1.1%; 95% CI: −1.3% to −0.9%; *p* < 0.001) with no absolute AAPC difference (*p* = 0.26). While the highest ASMR reduction in women occurred during 2001–2007 (APC = −2.2, *p* < 0.001), the largest reduction in men’s ASMRs was observed in an overlapping period, with different joinpoints, between 2002 and2006 (APC = −3.1, *p* < 0.001). There was a recent notable increase in women’s ASMRs from 2014 to 2019 (APC = 0.7; *p* < 0.001) (Figure 6 and Supplementary Table S7).

#### 3.4.3. Colorectal Cancer Age-Standardized Mortality Rate with Age Variation

Compared to older adults who had a decrease in ASMRs (AAPC = −1.4%; 95% CI: −1.6% to −1.2%; *p* < 0.001), younger adults had an overall increase in ASMRs (AAPC = 0.5%; 95% CI: 0.4–0.6%; *p* < 0.001) with an absolute AAPC difference of 1.9% (95% CI: 2.1–1.7%; *p* < 0.001) with non-parallel trends (*p* < 0.001), suggesting that ASMRs among younger adults are different and increasing at a greater rate than older adults (Figure 6 and Supplementary Table S7).

## 4. Discussion

Colorectal cancer (CRC) is the third most common diagnosis and second most common cause for cancer-related deaths in the US [18]. Our study highlights an overall decrease in CRC incidence, prevalence, and mortality over three decades. While the absolute number of CRC cases and number of deaths attributable to CRC increased in the US from 1990 to 2019, the ASIRs in the US showed a 12% decrease. In addition, there was an overall decrease in CRC age-standardized incidence, prevalence, and mortality rates for individuals aged 50–74 years, whereas those aged 15–49 years had a significant increase in these parameters. This trend was noticed in both genders. Overall, the lowest ASIRs and ASPRs were in many western states.

We found that there was an overall decrease in the incidence rate between 1990 and 2019. As screening became more widely implemented in the 1990s, an initial increase in CRC incidence was likely seen as a result of the improved detection of CRC cases [19]. However, an overall reduction in incidence is exemplified over the subsequent years, presumably due to endoscopic intervention at the time of colonoscopy, i.e., the removal of precancerous lesions [20]. A reverse in the trend is seen specifically in the age groups of 15–49 years, in which there was an overall increase in incidence rates over the past 30 years. Specifically, there was a notable increase in incidence during 1990–1994, followed by a slower rate growth in 1994–2017, and finally, a decrease in ASIRs in 2017–2019. The rising incidence of CRC among younger populations presents a complex interplay of various factors, as evidenced by the existing literature. Lifestyle contributors, including obesity, smoking, alcohol consumption, and occupational exposures, have been identified as potential risk factors [21,22,23]. These trends align with global patterns and suggest a multifaceted relationship between CRC incidence and changing societal behaviors [24]. Clinical observations in metastatic CRC highlight an increasing proportion of young adults, potentially related to the unique clinical and molecular features of CRC in this age group. Notably, the molecular distinctiveness observed in younger patients adds a layer of complexity to understanding CRC etiology in this demographic [25,26]. Ongoing research, such as that by Willauer et al. [25], sheds light on the distinct characteristics of CRC tumors in young adults, contributing valuable insights into the biological underpinnings of this concerning epidemiological shift.

Additionally, it appears there was a significant decrease in incidence most prominent in the east coast states. Meanwhile, the states that reported an increase in incidence were located primarily in the southern US. In fact, Mississippi was the only state with an increase in the CRC mortality rate when comparing data between 1990 and 2019. Along with other southern states, Mississippi saw an increase in both the incidence and prevalence of CRC. While the exact reason for this finding is unclear, a study published in 2011 noting the effects of socioeconomic factors on obesity rates in southern states, concluded that Mississippi had the highest rate of obesity, which could likely be a contributing factor to the high incidence of CRC [27]. Based on our data, the lowest CRC incidences were around the east and west coasts of the US in both 2009 and 2019. The discrepancy in the incidence seen in coastal states as compared to the south can potentially be multifactorial. Socioeconomic status could play a major role in this geographical discrepancy. Doubeni et al. showed that, compared to those with high socioeconomic status, people with the lowest socioeconomic status are 40% more likely to develop CRC [28]. Other factors include the prevalence of certain lifestyle habits such as smoking [21,29], alcohol use [22,30], and diet (including the consumption of red meat) [31,32,33] as well as obesity [23,34] and a lack of physical activity [35,36,37]. According to the CDC, obesity in adults largely plagues the southern US, and is well-known to correlate with dietary habits and physical activity [38]. The southern states, along with some midwestern states, including Missouri and Indiana, also have the highest smoking use in the US [39]. Risk factors such as family history of CRC and inflammatory bowel disease are less likely to explain the discrepancy between the southern and coastal US, as only approximately 1% of CRC cases can be attributed to these factors [40,41]. The same applies for inherited syndromes such as Lynch syndrome and Familial Adenomatous Polyposis, which account for only 5% of all CRC cases [18].

Mortality related to CRC followed a similar distribution as incidence between 1990 and 2019, with the highest rate noted in the southern United States and the lowest in California and New York. Overall death rates over the past 30 years decreased in both males and females. In the older population, mortality rates downtrended between 1990 and 2013, which can be attributed to advancements in and the utilization of screening colonoscopy and polypectomy. However, both genders saw an increase in mortality between 2013 and 2019. This may be secondary to the increasing prevalence of modifiable risk factors as discussed previously. Notably, mortality in the younger cohort aged 15–49 did increase overall between 1990 and 2019. This increase in mortality is one of the reasons for new colonoscopy screening guidelines by the American Cancer Society and the American College of Gastroenterology and the United States Preventive Services Task Force lowering the screening age to 45 years [42,43]. The overall decline in mortality is expected given the advancements in therapy and the increased availability of treatment for CRC. Current options include adjuvant chemotherapy for advanced cancer [18,44,45] and surgical intervention for localized carcinoma [46]. A study by Zeineddine et al. showed a substantial increase in the 5-year survival rate from 15.7% (2004–2006) to 26% (2013–2015), emphasizing the advancements in CRC management over the past two decades [47]. With frequent breakthroughs in chemotherapy and surgical techniques, we have seen a decline in colorectal-cancer-related mortality throughout the US, which is a trend that can be expected to continue.

In regard to the prevalence of CRC, there was an overall decrease in the US between 1990 and 2019. However, an increase in younger age groups between 1990 and 2019 was seen in comparison to the decreased prevalence in ages 50–74 years. This may be partially due to the increasing prevalence of obesity among all age groups in the United States [48,49,50]. Interestingly, a change in the trend was noted around 2014, with an increase in prevalence among older age groups and a decrease in the younger population. The change in prevalence could be attributed to an uptake in CRC screening secondary to the “80% by 2018” initiative. This initiative was launched by the National Colorectal Cancer Roundtable, an organization associated with the American Cancer Society and Center for Disease Control and Prevention, in an effort to screen 80% of recommended adults for colorectal cancer by 2018. According to Wender et al., multiple data sets across the US revealed rising CRC screening rates since the beginning of the campaign [48]. As discussed previously, an increase in screening results in more frequent identification of CRC, thereby explaining the rise prevalence.

This study possesses various noteworthy strengths. To the best of our knowledge, our study represents the most recent estimates of CRC prevalence, incidence, and mortality rates throughout the US population, specifically looking at these trends at the state level. Moreover, the major strength of this study is its comprehensive coverage over the last three decades, surpassing the scope of most previous studies, which estimated CRC epidemiology in limited geographical regions and for a shorter duration of time. In addition, this study provides valuable information and insights into the mortality rates attributed to CRC, an aspect of CRC that possesses relatively few studies in the existing literature. Several limitations should also be noted. Firstly, the study estimated rates of CRC as a whole, overlooking the potential differences in rates between specific types and stages of the disease. Secondly, the study could not determine the cause of increased mortality in CRC patients, thus making it difficult to suggest a course of corrective action. Moreover, the data itself could be subject to change due to modifications in disease classification or as a consequence of ongoing social unrest and rapid immigration between states. Therefore, careful interpretation of the findings is necessary, taking these data-related challenges into account. Lastly, our results are susceptible to ecological fallacy, and the absence of individual-level data restricts our capacity to draw individual-level inferences. Furthermore, we recognize the existence of potential confounding factors and variables that might interact with DALYs over time, such as lifestyle factors, comorbidities, access to healthcare, and treatment methods. It is essential to acknowledge that this limitation is intrinsic to the GBD study and beyond our control, but it must be taken into consideration when interpreting the findings.

## 5. Conclusions

In conclusion, developments in the screening programs, early detection, and treatment of CRC have resulted in overall decreased incidence, mortality, and prevalence in the US. Survival rates have improved with endoscopic intervention, including the removal of polyps, as well as screening modalities such as colonoscopy, fecal immunohistochemistry testing, and CT colonography. While these tools led to an initial increase in incidence and prevalence over the years by improving the detection of CRC, it was followed by a reduction in long-term mortality [51]. Trends in the US show that southern states, which have higher rates of obesity, tobacco use, limited physical activity, and generally poor lifestyle habits, have increased CRC incidence and mortality. However, advancements in treatment modalities have successfully improved the outcomes of CRC cases throughout the US. As noted between 1990 and 2019, lifestyle modifications, advancements in screening technology, and refinements in chemotherapy and surgical procedures will continue to impact the outcomes of CRC patients in upcoming years. Our data provide valuable information that may be helpful for US policymakers to provide new insights into the variability in CRC burden between states, to make better policy decisions, and to allocate appropriate resources for cancer prevention.

## Data Availability

Data are available in the supplementary file.

## Supplementary Materials

The following supporting information can be downloaded at: https://www.mdpi.com/article/10.3390/cancers16010205/s1, Table S1: Total and Age-Standardized Rate of All-Age, All-Colorectal Cancer Incidence, Prevalence, Mortality, DALYs, YLLs, and YLDs and their Percentage Change by US State in 1990 and 2019. Table S2: Total and Age-Standardized Rate of All-Age, All- Colorectal Cancer Incidence and Percentage Change of Incidence by US State in 1990 and 2019. Table S3: Total and Age-Standardized Rate of All-Age, All- Colorectal Cancer Prevalence and Percentage Change of Prevalence by US State in 1990 and 2019. Table S4: Total and Age-Standardized Rate of All-Age, All- Colorectal Cancer Mortality and Percentage Change of Mortality by US State in 1990 and 2019. Table S5: Trend analysis of CRC Age-Standardized Incidence rate with Gender and Age Variations from 1990 to 2019. Table S6: Trend analysis of CRC Age-Standardized Prevalence rate with Gender and Age Variations from 1990 to 2019. Table S7: Trend analysis of CRC Age-Standardized Mortality rate with Gender and Age Variations from 1990 to 2019.
