# Supplementary material for: The National Burden of Colorectal Cancer in the United States from 1990 to 2019
Source: Cancers (Basel). 2024 Jan 1;16(1):205. doi: 10.3390/cancers16010205 (PMC10778178; doi:10.3390/cancers16010205)
Supplement: Supplementary file 1 [file cancers-16-00205-s001.zip › cancers-2787581-supplementary.pdf]

Supplementary Tables

Title	Page
**Table S1**: Total and Age-Standardized Rate of All-Age, All-Colorectal Cancer Incidence, Prevalence, Mortality, DALYs, YLLs, and YLDs and their Percentage Change by US State in 1990 and 2019.	2
**Table S2:** Total and Age-Standardized Rate of All-Age, All- Colorectal Cancer Incidence and Percentage Change of Incidence by US State in 1990 and 2019	3-4
**Table S3:** Total and Age-Standardized Rate of All-Age, All- Colorectal Cancer Prevalence and Percentage Change of Prevalence by US State in 1990 and 2019	5-6
**Table S4:** Total and Age-Standardized Rate of All-Age, All- Colorectal Cancer Mortality and Percentage Change of Mortality by US State in 1990 and 2019	7-8
**Table S5:** Trend analysis of CRC Age-Standardized Incidence rate with Gender and Age Variations from 1990 to 2019	9-10
**Table S6:** Trend analysis of CRC Age-Standardized Prevalence rate with Gender and Age Variations from 1990 to 2019	10-11
**Table S7:** Trend analysis of CRC Age-Standardized Mortality rate with Gender and Age Variations from 1990 to 2019	11-12

	DALYs All ages Number 1990	DALYs All ages Number 2019	Change 2019/1990	DALYs Age-standardized rate 1990	DALYs Age-standardized rate 2019	Change 2019/1990
**Male**	714860 (693023, 733931)	980640 (941536, 1016252)	37.2%	531.9 (515.2, 546.6)	405.5 (389.5, 420.0)	-23.80%
**Female**	655179 (624754, 678379)	779999 (731006, 816467)	19.1%	371.1 (356.7, 382.8)	279.3 (264.5, 291.3)	-24.70%
**Both**	1370038 (1322424, 1408754)	1760640 (1681318, 1826205)	28.5%%	441.5 (427.0, 453.4)	338.9 (324.9, 350.8)	-23.20%
	**Deaths All ages Number 1990**	**Deaths All ages Number 2019**	**Change 2019/1990**	**Death Age-standardized rate 1990**	**Death Age-standardized rate 2019**	**Change 2019/1990**
**Male**	32008 (30831, 32897)	43826 (41775,45346)	36.90%	24.3 (23.3, 25.0)	17.5 (16.7, 18.1)	-27.80%
**Female**	33574 (31049, 35036)	40201 (36240, 42450)	19.70%	17.0(16.0, 17.7)	12.4 (11.4, 13.0)	-27.10%
**Both**	65582 (61888, 67693)	84026 (77987, 87516)	28.10%	20.0 (19.0, 20.7)	14.8 (13.9, 15.3)	-26.30%
	**Incidence All ages Number 1990**	**Incidence All ages Number 2019**	**Change 2019/1990**	**Incidence Age-standardized rate 1990**	**Incidence Age-standardized rate 2019**	**Change 2019/1990**
**Male**	77409 (75173, 79336)	122610 (99928, 148551)	58.40%	57.5 (55.7, 59.0)	49.4 (40.4, 59.9)	-14.10%
**Female**	75759 (71329, 78614)	104632 (86251, 125571)	38.10%	40.3 (38.3, 41.7)	35.2(28.9, 42.4)	-12.60%
**Both**	153168 (146891, 157265)	227242 (197022, 261375)	48.40%	47.6 (45.8, 48.9)	41.9 (36.1, 48.2)	-12.10%
	**Prevalence All ages Number 1990**	**Prevalence All ages Number 2019**	**Change 2019/1990**	**Prevalence Age-standardized rate 1990**	**Prevalence Age-standardized rate 2019**	**Change 2019/1990**
**Male**	434054 (420653, 447192)	745660 (615070, 899828)	71.80%	320.2 (310.0, 329.9)	302.2 (249.0, 364.4)	-5.60%
**Female**	432516 (412341, 450125)	631128 (529968, 751829)	45.90%	236.9 (227.3, 245.3)	221.0 (184.4, 264.1)	-6.70%
**Both**	866570 (834474, 896069)	1376788 (1200009, 1577837)	58.90%	273.3 (264.1, 281.9)	259.2 (225.2, 297.5)	-5.20%
	**YLDs All ages Number 1990**	**YLDs All ages Number 2019**	**Change 2019/1990**	**YLDs Age-standardized rate 1990**	**YLDs Age-standardized rate 2019**	**Change 2019/1990**
**Male**	40067 (30074, 51076)	65656 (44735, 90438)	63.90%	29.7 (22.2, 37.7)	26.7 (18.2, 36.8)	-10%
**Female**	38496 (28388, 49418)	54336 (38858, 72639)	41.10%	20.9 (15.3, 26.8)	18.9 (13.4, 25.4)	-9.50%
**Both**	78563 (58665, 100407)	119992 (85825, 157158)	52.70%	24.7 (18.4, 31.5)	22.6 (16.1, 29.6)	-8.60%
	**YLLs All ages Number 1990**	**YLLs All ages Number 2019**	**Change 2019/1990**	**YLLs Age-standardized rate 1990**	**YLLs Age-standardized rate 2019**	**Change 2019/1990**
**Male**	674793 (657157, 689445)	914984 (885600, 940480)	35.60%	502.3 (488.7, 513.3)	378.8 (366.8, 389.5)	-24.60%
**Female**	616683 (588371, 635993)	725664 (683336, 755087)	17.70%	350.3 (337.3, 360.1)	260.4 (247.8, 269.6)	-25.70%
**Both**	1291476 (1249198, 1320455)	1640648 (1574853, 1689210)	27%	416.8 (404.6, 425.7)	316.3 (304.9, 324.9)	-24.10%

Table S1: Total and Age-Standardized Rate of All-Age, All–Colon and Rectal Cancer Incidence, Prevalence, Mortality, DALYs, YLLs, and YLDs and their Percentage Change by US State in 1990 and 2019.

Abbreviations: DALYs: disability-adjusted life years, YLDs: years lived with disability, YLLs: years of life lost.

State	Age-standardized Incidence rate 1990			Age-standardized Incidence rate 2019			Change<br><br>2019/1990	Number of Incidence 1990			Number of Incidence 1990			Change 2019/1990
	Both	Upper	Lower	Both	Upper	Lower		Both	Upper	Lower	Both	Upper	Lower	
Alabama	43.5	41.2	45.8	44.7	35.6	55.5	2.8%	2329	2205	2453	3705	2949	4571	59.0%
Alaska	46.1	42.6	49.5	44.2	35.6	54.0	-4.2%	133	124	143	459	369	566	245.8%
Arizona	41.2	38.5	43.6	38.5	30.3	47.8	-6.7%	1954	1819	2069	4682	3715	5794	139.6%
Arkansas	43.8	41.4	46.1	44.3	35.1	55.3	1.1%	1513	1426	1596	2247	1791	2785	48.5%
California	39.7	37.5	41.4	38.3	30.0	47.9	-3.4%	13100	12374	13676	23497	18484	29296	79.4%
Colorado	42.7	39.8	45.6	38.5	30.6	48.6	-9.8%	1520	1416	1621	3278	2603	4120	115.6%
Connecticut	49.4	46.4	51.9	38.2	30.2	47.8	-22.8%	2280	2140	2401	2574	2042	3200	12.9%
Delaware	52.6	49.1	56.2	40.7	33.1	48.9	-22.5%	438	409	468	715	582	858	63.2%
District of Columbia	46.8	43.9	49.7	39.8	31.4	49.4	-14.8%	384	358	408	354	278	436	-8.0%
Florida	44.9	42.3	47.2	41.1	32.9	51.2	-8.4%	10027	9392	10582	16597	13354	20441	65.5%
Georgia	39.1	37.2	41.0	42.8	34.3	53.4	9.4%	2745	2608	2883	6718	5360	8408	144.8%
Hawaii	44.6	41.6	47.6	42.4	33.7	53.1	-4.8%	578	541	619	1137	909	1407	96.6%
Idaho	40.0	37.2	42.8	36.6	29.0	45.3	-8.4%	496	460	533	1010	801	1250	103.6%
Illinois	51.5	48.8	54.0	43.6	34.2	54.3	-15.4%	7640	7222	8020	9301	7336	11556	21.7%
Indiana	50.6	47.7	53.2	44.2	35.0	54.4	-12.6%	3639	3421	3826	4847	3853	5942	33.2%
Iowa	46.5	43.6	49.1	43.6	34.7	54.0	-6.3%	2024	1885	2147	2436	1952	3001	20.4%
Kansas	47.2	44.2	50.0	42.4	33.5	52.3	-10.3%	1631	1517	1730	2044	1616	2522	25.3%
Kentucky	56.5	53.3	59.5	55.3	44.4	67.3	-2.1%	2720	2560	2869	4142	3336	5021	52.3%
Louisiana	49.5	46.8	51.9	54.4	43.6	68.0	9.9%	2433	2300	2554	3931	3154	4900	61.5%
Maine	54.5	50.6	58.0	43.1	34.2	52.9	-20.9%	920	853	980	1196	951	1464	30.0%
Maryland	54.6	51.7	57.5	42.5	33.5	52.4	-22.3%	3014	2856	3174	4205	3336	5174	39.5%
Massachusetts	61.4	57.8	64.7	41.2	33.2	50.5	-32.9%	5146	4823	5441	4871	3930	5949	-5.3%
Michigan	45.8	43.5	47.9	40.8	32.1	50.1	-11.1%	5310	5030	5552	7131	5645	8766	34.3%
Minnesota	53.6	50.5	57.0	40.7	32.6	50.7	-24.1%	3028	2824	3217	3846	3089	4749	27.0%
Mississippi	40.6	38.3	43.0	46.7	37.0	58.2	14.8%	1323	1242	1400	2241	1780	2775	69.4%
Missouri	49.2	46.6	51.9	42.5	33.7	52.3	-13.6%	3580	3369	3784	4541	3587	5588	26.8%
Montana	44.3	41.0	47.8	39.6	32.1	48.2	-10.5%	480	443	517	775	627	941	61.6%
Nebraska	51.3	48.2	54.5	43.8	34.9	54.7	-14.7%	1149	1074	1226	1385	1109	1726	20.5%
Nevada	50.3	47.2	53.4	45.3	36.3	56.0	-9.9%	642	601	682	2225	1778	2758	246.8%
New Hampshire	55.2	51.1	59.0	42.8	34.1	52.6	-22.4%	732	675	783	1085	864	1330	48.3%
New Jersey	58.3	54.9	61.2	46.3	37.0	57.8	-20.5%	6226	5852	6536	7189	5792	8925	15.5%
New Mexico	34.8	32.6	36.9	38.8	30.4	49.1	11.6%	595	557	630	1439	1125	1807	141.9%
New York	49.6	47.1	52.2	38.4	30.1	47.8	-22.6%	12184	11520	12848	13001	10228	16101	6.7%

North Carolina	42.2	40.0	44.4	40.5	32.6	49.8	-4.1%	3509	3319	3691	7000	5668	8614	99.5%
North Dakota	48.5	44.9	51.8	43.9	36.0	54.2	-9.5%	438	404	469	533	439	657	21.6%
Ohio	53.2	50.4	55.9	44.3	35.3	54.4	-16.8%	7687	7251	8086	9036	7176	11058	17.5%
Oklahoma	43.8	41.3	46.3	43.0	34.2	53.1	-1.6%	1898	1779	2012	2718	2167	3339	43.2%
Oregon	46.0	43.2	48.6	40.9	32.6	51.1	-11.0%	1801	1688	1908	3003	2403	3731	66.8%
Pennsylvania	55.4	52.4	58.2	45.5	35.8	57.6	-17.8%	10128	9523	10673	10921	8700	13841	7.8%
Rhode Island	55.7	52.0	59.6	42.4	33.8	52.4	-23.9%	833	772	894	810	647	1000	-2.8%
South Carolina	39.9	37.5	42.0	41.6	33.3	50.9	4.3%	1636	1541	1726	3571	2871	4361	118.2%
South Dakota	45.8	42.3	49.2	43.0	34.6	52.5	-6.1%	465	430	501	639	518	772	37.3%
Tennessee	44.1	41.8	46.3	43.6	34.8	54.0	-1.0%	2837	2681	2984	4911	3912	6065	73.1%
Texas	43.8	41.5	45.9	41.5	32.7	51.8	-5.1%	8008	7590	8405	16370	12882	20405	104.4%
Utah	34.4	32.1	36.5	36.4	28.9	45.2	5.8%	544	508	577	1376	1092	1708	152.6%
Vermont	47.9	44.4	51.1	43.9	36.0	52.8	-8.3%	332	307	355	545	447	654	64.4%
Virginia	50.3	47.6	52.9	43.1	33.8	53.7	-14.4%	3546	3351	3737	6001	4731	7427	69.2%
Washington	42.5	40.1	44.5	37.7	29.7	47.2	-11.3%	2525	2380	2654	4574	3632	5713	81.1%
West Virginia	46.3	43.6	49.0	48.5	38.4	60.2	4.8%	1263	1186	1340	1752	1392	2177	38.7%
Wisconsin	54.0	51.1	56.9	41.8	32.9	52.0	-22.6%	3590	3381	3796	4301	3398	5324	19.8%
Wyoming	42.3	39.0	45.4	37.5	31.0	44.4	-11.4%	213	196	229	376	312	447	76.7%
United States of America	47.6	45.8	48.9	41.9	36.1	48.2	-12.1%	153168	146891	157265	227242	197022	261375	48.4%

Table S2: Total and Age-Standardized Rate of All-Age, All- Colon and Rectal Cancer Incidence and Percentage Change of Incidence by US State in 1990 and 2019

State	Age-standardized Prevalence rate 1990			Age-standardized Prevalence rate 2019			Change 2019/1990	Number of Prevalence 1990			Number of Prevalence 1990			Change 2019/1990
	**Both**	**Upper**	**Lower**	**Both**	**Upper**	**Lower**		**Both**	**Upper**	**Lower**	**Both**	**Upper**	**Lower**	
Alabama	240.0	227.3	254.2	259.3	208.2	320.8	8.0%	12652	11960	13435	21062	17014	25972	66.5%
Alaska	248.5	230.8	267.4	271.2	219.9	329.9	9.1%	787	729	848	2869	2317	3517	264.4%
Arizona	242.8	227.5	258.4	241.0	192.1	298.1	-0.7%	11436	10663	12212	28634	22983	35259	150.4%
Arkansas	245.9	232.0	261.0	258.7	206.9	320.8	5.2%	8276	7803	8790	12808	10264	15783	54.8%
California	226.0	214.2	236.9	245.5	195.2	302.7	8.6%	74281	70264	77952	147728	118058	181114	98.9%
Colorado	252.0	235.4	269.3	242.0	193.4	302.6	-4.0%	8947	8339	9553	20441	16298	25523	128.5%
Connecticut	285.9	269.1	301.9	241.6	193.7	299.4	-15.5%	12986	12190	13736	15774	12672	19473	21.5%
Delaware	291.7	273.3	312.2	250.7	206.2	298.4	-14.0%	2426	2271	2601	4309	3532	5132	77.7%
District of Columbia	225.6	210.5	240.8	237.0	190.1	291.8	5.0%	1803	1685	1931	2057	1653	2518	14.1%
Florida	265.9	251.3	280.5	261.0	210.1	321.5	-1.8%	58062	54398	61635	101569	82276	124712	74.9%
Georgia	214.8	203.1	226.7	255.4	205.7	318.9	18.9%	15034	14219	15909	40078	32210	50101	166.6%
Hawaii	273.1	256.2	290.8	275.0	221.1	340.0	0.7%	3552	3329	3785	7128	5788	8763	100.7%
Idaho	233.5	217.7	251.7	225.1	181.3	276.8	-3.6%	2864	2662	3088	6121	4913	7550	113.7%
Illinois	290.5	275.2	306.0	271.0	216.1	335.2	-6.7%	42349	40063	44668	56482	45053	69991	33.4%
Indiana	289.7	272.3	305.4	267.3	212.4	328.1	-7.8%	20520	19243	21699	28712	22839	35117	39.9%
Iowa	272.2	255.5	287.1	267.8	213.9	331.7	-1.6%	11476	10744	12162	14390	11588	17737	25.4%
Kansas	282.4	264.8	299.9	261.7	209.0	322.4	-7.3%	9459	8840	10074	12284	9833	15072	29.9%
Kentucky	331.1	312.1	350.8	333.9	269.6	405.6	0.9%	15672	14736	16642	24600	19922	29649	57.0%
Louisiana	284.0	268.4	299.7	331.3	266.4	410.6	16.6%	13830	13070	14626	23598	18964	29184	70.6%
Maine	317.1	295.5	338.3	263.3	211.9	321.8	-17.0%	5246	4873	5609	7128	5773	8657	35.9%
Maryland	304.7	287.6	322.5	263.4	210.0	323.5	-13.6%	16818	15855	17797	25673	20424	31594	52.7%
Massachusetts	361.8	342.3	382.5	263.2	214.2	319.5	-27.3%	29626	27846	31438	30158	24570	36407	1.8%
Michigan	258.4	245.4	271.2	246.8	198.2	302.9	-4.5%	29687	28178	31196	42090	33903	51535	41.8%
Minnesota	325.8	306.4	346.2	259.7	210.4	319.9	-20.3%	17903	16762	19084	23915	19368	29512	33.6%
Mississippi	222.2	208.9	236.0	265.4	212.5	329.7	19.5%	7084	6643	7543	12521	10037	15472	76.7%
Missouri	282.5	267.4	299.8	259.5	208.3	319.0	-8.2%	20011	18888	21293	27008	21713	33093	35.0%
Montana	256.8	237.3	276.8	240.8	196.1	291.3	-6.3%	2742	2525	2957	4595	3739	5567	67.6%
Nebraska	304.4	285.8	324.9	272.1	219.5	336.5	-10.6%	6592	6167	7025	8349	6778	10295	26.6%
Nevada	271.1	252.9	287.9	272.6	218.4	335.5	0.6%	3659	3416	3897	13388	10731	16527	265.9%
New Hampshire	320.5	297.8	343.2	267.1	213.8	326.6	-16.7%	4196	3899	4500	6663	5353	8135	58.8%
New Jersey	337.4	317.5	356.0	294.8	237.9	361.3	-12.6%	35594	33468	37590	44460	35922	54552	24.9%

New Mexico	198.1	185.5	211.6	233.0	185.4	292.6	17.6%	3382	3168	3606	8424	6707	10484	149.1%
New York	280.4	265.2	295.7	247.1	196.3	305.6	-11.9%	67620	63802	71564	81128	65305	99781	20.0%
North Carolina	237.9	224.7	251.0	245.9	198.0	302.4	3.4%	19675	18578	20809	41935	33878	51549	113.1%
North Dakota	287.3	267.0	307.9	271.5	224.3	333.2	-5.5%	2516	2329	2710	3172	2624	3897	26.1%
Ohio	303.5	287.6	320.1	266.4	213.2	325.3	-12.2%	43231	40930	45716	52904	42469	64306	22.4%
Oklahoma	252.5	237.0	267.5	254.2	204.9	315.5	0.7%	10694	10024	11338	15719	12740	19268	47.0%
Oregon	273.3	256.0	290.3	256.6	207.0	316.5	-6.1%	10542	9865	11230	18420	14919	22707	74.7%
Pennsylvania	315.9	298.1	333.4	280.5	222.4	352.5	-11.2%	56623	53148	59969	64940	51602	81352	14.7%
Rhode Island	321.6	300.6	345.9	265.7	214.8	325.5	-17.4%	4693	4367	5053	4916	3986	6034	4.7%
South Carolina	218.8	205.9	232.1	246.8	198.6	300.4	12.8%	8970	8430	9527	20871	16847	25303	132.7%
South Dakota	269.3	249.9	288.3	262.4	214.1	318.0	-2.5%	2640	2452	2836	3747	3067	4507	41.9%
Tennessee	246.9	234.2	261.2	258.8	208.3	320.2	4.8%	15664	14833	16615	28637	23093	35354	82.8%
Texas	251.4	237.8	264.4	255.1	203.7	316.0	1.5%	45715	43150	48086	99954	79701	123683	118.6%
Utah	203.0	189.2	215.9	224.5	179.5	278.1	10.6%	3208	2988	3418	8445	6754	10465	163.3%
Vermont	272.3	252.1	291.5	274.6	226.0	329.9	0.8%	1856	1720	1988	3331	2751	3990	79.5%
Virginia	288.1	271.8	305.3	268.7	214.4	333.0	-6.7%	20311	19135	21520	36989	29486	45574	82.1%
Washington	249.8	236.6	262.9	235.8	188.5	292.4	-5.6%	14732	13930	15549	28174	22546	34962	91.2%
West Virginia	257.7	242.2	275.0	280.0	224.9	345.9	8.7%	6890	6450	7379	9846	7979	12126	42.9%
Wisconsin	320.4	301.9	339.3	262.9	208.0	326.2	-18.0%	20808	19519	22050	26373	20936	32571	26.7%
Wyoming	244.9	226.7	264.7	229.1	191.4	271.5	-6.5%	1229	1137	1328	2265	1892	2685	84.3%
United States of America	273.3	264.1	281.9	259.2	225.2	297.5	-5.2%	866570	834474	896069	1376788	1200009	1577837	58.9%

Table S3: Total and Age-Standardized Rate of All-Age, All- Colon and Rectal Cancer Prevalence and Percentage Change of Prevalence by US State in 1990 and 2019

State	Age-standardized Mortality rate 1990			Age-standardized Mortality rate 2019			Change 2019/1990	Number of Mortality 1990			Number of Mortality 1990			Change 2019/1990
	Both	Upper	Lower	Both	Upper	Lower		Both	Upper	Lower	Both	Upper	Lower	
Alabama	20.3	19.2	21.3	18.4	15.5	21.7	-9.2%	1109	1044	1162	1579	1321	1852	42.4%
Alaska	22.2	20.4	23.7	15.8	13.7	18.1	-28.8%	55	52	59	158	137	182	185.3%
Arizona	16.9	15.8	17.9	13.2	11.1	15.6	-21.9%	804	750	850	1696	1431	1983	110.9%
Arkansas	19.9	18.7	20.9	18.0	15.3	21.2	-9.5%	712	668	750	956	811	1119	34.2%
California	17.4	16.4	18.1	12.4	10.4	14.5	-28.8%	5757	5419	6008	7948	6702	9319	38.1%
Colorado	17.2	16.1	18.3	13.1	10.8	15.4	-24.3%	615	573	654	1127	935	1329	83.4%
Connecticut	20.1	18.9	21.1	12.9	10.6	15.3	-36.0%	949	888	998	935	773	1103	-1.5%
Delaware	23.2	21.7	24.6	14.6	12.7	16.7	-37.2%	193	181	204	267	232	307	38.4%
District of Columbia	27.8	26.1	29.5	16.5	14.0	19.3	-40.9%	241	222	256	154	130	180	-36.1%
Florida	18.1	16.9	18.9	13.7	11.6	16.2	-23.9%	4178	3900	4391	6062	5108	7109	45.1%
Georgia	19.2	18.2	20.0	17.0	14.4	19.9	-11.6%	1347	1276	1406	2665	2257	3128	97.9%
Hawaii	16.9	15.7	17.9	13.5	11.2	15.9	-19.9%	217	202	230	395	330	461	82.3%
Idaho	16.6	15.3	17.7	13.1	11.1	15.4	-21.3%	209	193	223	371	313	434	77.7%
Illinois	22.3	21.1	23.3	15.2	12.8	18.0	-31.8%	3388	3186	3545	3426	2867	4056	1.1%
Indiana	21.1	19.8	22.1	16.5	13.8	19.4	-21.8%	1545	1449	1617	1876	1574	2201	21.4%
Iowa	18.7	17.4	19.7	15.5	13.0	18.3	-17.4%	850	783	899	935	790	1102	10.0%
Kansas	18.2	16.9	19.2	14.9	12.5	17.5	-17.8%	662	609	700	764	637	898	15.4%
Kentucky	21.7	20.4	22.9	19.5	16.4	22.8	-10.2%	1072	999	1129	1506	1266	1755	40.4%
Louisiana	20.8	19.5	21.7	19.5	16.3	22.8	-6.4%	1034	971	1081	1448	1214	1693	40.1%
Maine	21.3	19.7	22.6	15.4	13.1	17.8	-28.0%	372	341	395	447	379	517	20.3%
Maryland	23.8	22.5	24.9	15.2	12.6	17.8	-36.4%	1308	1237	1369	1554	1279	1817	18.8%
Massachusetts	23.0	21.5	24.1	13.3	11.2	15.6	-42.1%	1998	1855	2101	1691	1423	1986	-15.4%
Michigan	19.7	18.6	20.5	15.1	12.7	17.8	-23.0%	2291	2166	2391	2780	2338	3253	21.3%
Minnesota	19.0	17.7	20.0	13.1	11.0	15.4	-30.8%	1124	1034	1187	1316	1102	1539	17.1%
Mississippi	19.8	18.7	20.8	20.1	17.2	23.4	1.8%	661	622	695	995	848	1158	50.5%
Missouri	20.6	19.4	21.6	15.5	13.0	18.3	-24.8%	1560	1455	1641	1740	1454	2062	11.6%
Montana	18.5	17.1	19.9	14.6	12.4	17.1	-21.2%	204	188	219	299	255	350	46.7%
Nebraska	19.8	18.5	21.0	15.1	12.9	17.8	-23.9%	471	436	500	511	435	596	8.4%
Nevada	23.5	22.1	24.8	16.9	14.4	19.8	-28.0%	271	257	286	826	703	967	204.8%
New Hampshire	21.5	19.9	22.9	14.4	12.0	16.7	-33.0%	290	268	309	377	315	438	29.9%
New Jersey	23.5	22.0	24.6	15.3	13.0	18.1	-34.6%	2542	2385	2668	2552	2149	2998	0.4%
New Mexico	16.4	15.2	17.3	15.4	13.1	18.0	-5.5%	278	259	295	603	510	701	116.4%
New York	21.7	20.4	22.7	12.7	10.5	15.0	-41.4%	5471	5128	5732	4631	3814	5461	-15.3%

North Carolina	18.9	17.8	19.7	15.0	12.6	17.7	-20.9%	1571	1475	1638	2657	2250	3134	69.1%
North Dakota	19.1	17.7	20.4	15.4	13.2	17.6	-19.6%	182	167	195	204	173	234	11.8%
Ohio	22.2	20.9	23.2	16.6	14.1	19.3	-24.9%	3259	3057	3411	3577	3029	4117	9.8%
Oklahoma	18.4	17.3	19.5	17.0	14.4	19.9	-7.6%	829	776	876	1117	941	1305	34.8%
Oregon	17.7	16.5	18.6	13.6	11.5	15.9	-23.1%	706	659	744	1043	880	1222	47.7%
Pennsylvania	23.0	21.6	24.0	16.0	13.5	19.0	-30.1%	4295	4019	4494	4139	3481	4879	-3.6%
Rhode Island	22.4	20.7	23.9	14.3	12.1	16.8	-36.2%	348	319	373	294	247	344	-15.5%
South Carolina	19.2	18.0	20.1	16.3	13.6	19.3	-15.1%	781	735	820	1432	1197	1696	83.3%
South Dakota	18.9	17.4	20.1	15.9	13.9	18.4	-15.5%	204	186	217	256	222	293	25.7%
Tennessee	19.8	18.6	20.7	16.9	14.3	19.8	-14.4%	1291	1218	1355	1957	1658	2292	51.5%
Texas	18.6	17.5	19.3	15.0	12.5	17.4	-19.5%	3424	3223	3566	5970	4984	6941	74.4%
Utah	14.6	13.6	15.4	13.1	11.1	15.2	-10.3%	230	214	243	499	424	581	117.1%
Vermont	20.5	19.0	21.8	14.7	12.9	16.7	-28.2%	146	134	155	192	168	218	32.2%
Virginia	20.9	19.7	21.9	14.7	12.3	17.3	-29.4%	1465	1384	1536	2105	1751	2460	43.7%
Washington	16.9	15.8	17.7	12.8	10.7	15.0	-24.2%	1010	947	1061	1602	1343	1877	58.7%
West Virginia	20.8	19.5	22.0	19.6	16.4	22.9	-6.0%	582	542	616	738	620	862	27.0%
Wisconsin	20.2	18.9	21.2	13.9	11.7	16.4	-31.3%	1393	1297	1463	1512	1271	1783	8.6%
Wyoming	17.9	16.5	19.3	13.9	12.2	15.6	-22.7%	91	83	97	144	126	162	58.7%
United States of America	20.0	19.0	20.7	14.8	13.9	15.3	-26.3%	65582	61888	67693	84026	77987	87516	28.1%

Table S4: Total and Age-Standardized Rate of All-Age, All- Colon and Rectal Cancer Mortality and Percentage Change of Mortality by US State in 1990 and 2019

Incidence	Trends[a]			Gender/Age-specific AAPC difference (95% CI)[b]	Pairwise comparison P-values		
	Time period	APC (95% CI)	AAPC (95% CI)		Gender/Age-specific AAPC difference	Coincidence[c]	Parallelism[d]
**Gender**							
Male	1990-1994	1.1 (0.2 to 2.0)	-0.5 (-0.8 to -0.2)	0	0.126	<0.0001	<0.0001
	1994-2002	-0.7 (-1.1 to -0.4)					
	2002-2006	-2.9 (-4.2 to -1.5)					
	2006-2015	-0.4 (-0.7 to -0.1)					
	2015-2019	0.7 (-0.3 to 1.6)					
Female	1990-1994	0.9 (0.1 to 1.8)	-0.5 (-0.7 to -0.3)				
	1994-2002	-0.1 (-0.5 to 0.2)					
	2002-2006	-2.8 (-4.0 to -1.5)					
	2006-2019	-0.4 (-0.6 to -0.3)					
**Age**							
50-74 years	1990-1994	0.8 (0.1 to 1.6)	-0.6 (-0.9 to -0.4)	-1.8 (-2.4 - -1.2)	<0.001	<0.0001	<0.0001
	1994-2002	-1.8 (-2.1 to -1.5)					
	2002-2006	-3.5 (-4.6 to -2.4)					
	2006-2014	-0.0 (-0.3 to 0.3)					
	2014-2019	1.5 (0.9 to 2.0)					
15-49 years	1990-1994	4.1 (2.8 to 5.6)	1.2 (0.9 to 1.6)				
	1994-2002	2.4 (1.8 to 3.0)					
	2002-2017	0.5 (0.3 to 0.7)					
	2017-2019	-3.2 (-7.3 to -1.0)					

Table S5: Age and gender-Specific Trends for Colon and Rectal Cancer (CRC) Incidence Among Different Age and Gender Groups

Abbreviations: APC: annual percentage change , AAPC: average annual percentage change.

[a] Time-trends were computed using Joinpoint Regression Program (v5.0.2, NCI) with 4 maximum joinpoints allowed (5-line segments).
[b] A negative value indicates a greater AAPC in younger compared to older age group.

[c] Tests whether age and gender-specific trends were identical. A significant P-value indicates that the trends were not identical (i.e., they had different incidence rates and coincidence was rejected).
[d] Tests whether race-specific trends were parallel. A significant P-value indicates that the trends were not parallel (i.e., parallelism was rejected)

**Prevalence**	Trends[a]			Gender-specific AAPC difference (95% CI)[b]	Pairwise comparison P-values		
	Time period	APC (95% CI)	AAPC (95% CI)		Gender-specific AAPC difference	Coincidence[c]	Parallelism[d]
Gender							
Male	1990-1994	1.5 (1.3 to 1.7)	-0.2 (-0.3 to -0.1)	0	<0.188	<0.001	<0.001
	1994-1997	-0.2 (-0.8 to 0.5)					
	1997-2001	-0.7 (-1.1 to -0.4)					
	2001-2005	-2.0 (-2.3 to -1.7)					
	2005-2015	-0.3 (-0.3 to -0.2)					
	2015-2019	0.6 (0.4 to 0.8)					
Female	1990-1994	0.9 (0.1 to 1.8)	-0.2 (-0.3 to -0.2)				
	1994-2000	-0.1 (-0.5 to 0.2)					
	2000-2006	-2.8 (-4.0 to -1.5)					
	2006-2015	-0.4 (-0.6 to -0.3)					
	2015-2019	0.1 (0.0 to 0.2)					
Age							
50-74 years	1990-1993	1.3 (1.0 to 1.6)	-0.4 (-0.5 to -0.3)	-1.7	7<0.001	<0.001	<0.001
	1993-1996	-0.3 (-0.9 to 0.2)					
	1996-2000	-1.9 (-2.2 to -1.6)					
	2000-2006	-2.4 (-2.6 to -2.3)					
	2006-2014	0.0 (-0.0 to 0.1)					
	2014-2019	1.4 (1.3 to 1.6)					
	1990-1995	4.2 (3.9 to 4.4)	1.3 (1.2 to 1.5)				

15-49 years	1995-2001	2.0 (1.8 to 2.3)					
	2001-2010	1.0 (0.9 to 1.2)					
	2010-2014	-0.1 (-0.6 to 0.4)					
	2014-2017	0.6 (-0.4 to 1.7)					
	2017-2019	-2.6 (-3.6 to -1.6)					

Table S6: Age and gender-Specific Trends for Colon and Rectal Cancer (CRC) Prevalence: Among Different Age and Gender Groups

[a] Time-trends were computed using Joinpoint Regression Program (v5.0.2, NCI) with 4 maximum joinpoints allowed (5-line segments).
[b] A negative value indicates a greater AAPC in younger compared to older age group.
[c] Tests whether age and gender-specific trends were identical. A significant P-value indicates that the trends were not identical (i.e., they had different prevalence rates and coincidence was rejected).
[d] Tests whether race-specific trends were parallel. A significant P-value indicates that the trends were not parallel (i.e., parallelism was rejected).

**Mortality**	Trends[a]			Gender/Age-specific AAPC difference (95% CI)[b]	Pairwise comparison P-values		
	Time period	APC (95% CI)	AAPC (95% CI)		Gender/Age-specific AAPC difference	Coincidence[c]	Parallelism[d]
Gender							
Male	1990-1995	-0.7 (-1.1 to -0.4)	-1.1 (-1.3 to -0.9)	0	<0.246	<0.001	<0.001
	1995-1998	-2.1 (-3.7 to -0.5)					
	1998-2002	-1.1 (-1.9 to -0.2)					
	2002-2006	-3.1 (-3.9 to -2.3)					
	2006-2014	-1.1 (-1.3 to -0.9)					
	2014-2019	0.6 (0.3 to 1.0)					
Female	1990-2001	-0.9 (-1.0 to -0.7)	-1.1 (-1.2 to -0.9)				
	2001-2007	-2.2 (-2.7 to -1.8)					
	2007-2013	-1.2 (-1.7 to -0.8)					
	2013-2019	-0.2 (-0.5 to 0.2)					

Age							
50-74 years	1990-1995	-1.3 (-1.8 to -0.8)	-1.4 (-1.5 to -1.2)	-1.9	1. <0.0017	<0.001	<0.001
	1995-2007	-3.1 (-3.2 to -2.9)					
	2007-2013	-0.7 (-1.2 to -0.3)					
	2013-2019	1.5 (1.1 to 1.9)					
15-49 years	1990-1995	2.3 (1.6 to 2.9)	0.5 (0.0 to 0.9)				
	1995-1998	-0.0 (-2.9 to 2.9)					
	1998-2001	2.7 (-0.2 to 5.7)					
	2001-2017	-0.0 (-0.2 to 0.1)					
	2017-2019	-2.5 (-5.2 to 0.4)					

Table S7: Age and gender-Specific Trends for Colon and Rectal Cancer (CRC) Mortality: Among Different Age and Gender Groups.

Abbreviations: APC: annual percentage change, AAPC: average annual percentage change.

[a] Time-trends were computed using Joinpoint Regression Program (v5.0.2, NCI) with 4 maximum joinpoints allowed (5-line segments).
[b] A negative value indicates a greater AAPC in younger compared to older age group.
[c] Tests whether age and gender-specific trends were identical. A significant P-value indicates that the trends were not identical (i.e., they had different mortality rates and coincidence was rejected).
[d] Tests whether race-specific trends were parallel. A significant P-value indicates that the trends were not parallel (i.e., parallelism was rejected).
